# Low Transfer of Tacrolimus and Its Metabolites into Colostrum of Graft Recipient Mothers

**DOI:** 10.3390/nu10030267

**Published:** 2018-02-27

**Authors:** Bozena Kociszewska-Najman, Natalia Mazanowska, Bronislawa Pietrzak, Leszek Paczek, Monika Szpotanska-Sikorska, Joanna Schreiber-Zamora, Ewa Hryniewiecka, Dorota Zochowska, Emilia Samborowska, Michal Dadlez, Miroslaw Wielgos

**Affiliations:** 1 First Department of Obstetrics and Gynecology, Medical University of Warsaw, 02-015 Warsaw, Poland; bnajman@wum.edu.pl (B.K.-N.); bpietrzak@wum.edu.pl (B.P.); mszpotanska@wp.pl (M.S.-S.); jzamora1@wp.pl (J.S.-Z.); miroslaw.wielgos@wum.edu.pl (M.W.); 2 Department of Immunology, Transplant Medicine and Internal Diseases, Transplantation Institute, Medical University of Warsaw, 00-001 Warsaw, Poland; leszek.paczek@wum.edu.pl (L.P.); elhryniewiecka@gmail.com (E.H.); d_zochowska@wp.pl (D.Z.); 3 Department of Bioinformatics, Institute of Biochemistry and Biophysics, Polish Academy of Sciences, 02-106 Warsaw, Poland; 4 Mass Spectrometry Laboratory, Institute of Biochemistry and Biophysics, Polish Academy of Sciences, 02-106 Warsaw, Poland; emi.sambor@gmail.com (E.S.); michald@ibb.waw.pl (M.D.); 5 Institute of Genetics and Biotechnology, Biology Department, Warsaw University, 02-096 Warsaw, Poland

**Keywords:** transplantation, breastfeeding, HPLC, drug safety, drug metabolism

## Abstract

Currently, the majority of neonates born to organ recipient mothers on chronic immunosuppressive therapy are formula fed. However, over the past few years, evidence has grown, suggesting that breastfeeding might be possible and beneficial. We designed a study assessing the transfer of tacrolimus into the colostrum of posttransplant mothers. We assessed the amount of tacrolimus and its metabolites, M-1 and M-3, that would be ingested by the breastfed neonates. Concentrations of tacrolimus and its metabolites were measured in colostrum from 14 posttransplant mothers as well as in venous cord blood and venous blood of the neonates. Test material analysis was performed by liquid chromatography coupled with mass spectrometry (LC/MS). The amount of ingested formula was registered, which allowed for estimation of the amount of tacrolimus and its metabolites that would be ingested by breastfed infants. The mean amount of tacrolimus that would be ingested by the neonates in maternal milk was 151.4 ng/kg/24 h (standard deviation SD ± 74.39); metabolite M-1: 23.80 ng/kg/24 h (SD ± 14.53); and metabolite M-3: 13.25 ng/kg/24 h (SD ± 9.05). The peak level of tacrolimus and metabolite M-1 in colostrum was noted 8 h after an oral dose (3.219 ng/mL SD ± 2.22 and 0.56 ng/mL SD ± 0.60, respectively) and metabolite M-3 after 6 h (0.29 ng/mL SD ± 0.22). Low concentrations of tacrolimus and its metabolites, M-1 and M-3, in colostrum show that neonates will ingest trace amounts of the drug. Further studies are required to fully assess the safety of breastfeeding by posttransplant mothers.

## 1. Introduction

The pregnancy and delivery by an organ recipient mother is a high-risk one. The effect of the chronic immunosuppressant drugs used during pregnancy on the developing organs of fetuses and their effect on their future life remains unclear, and the literature reports are still scarce. The same applies to the effect of potential breastfeeding. Breastfeeding is a perfectly adapted manner of supplying nutrition to the infant and is essential to the achievement of optimal child health. Mother’s milk transmits elements of microbiome and immune responses and provides specific probiotics and prebiotics that support growth of beneficial bacteria. Evidence also exists that multipotential stem cells are secreted into breast milk and can persist within infants [1,2,3,4]. According to the American Academy of Pediatrics, breast milk is the superior form of nutrition for an infant through the first 12 months, especially for preterm and low-birth-weight (LBW) infants. New findings show that breastfeeding is potentially one of the top interventions for reducing mortality in preterm infants [5,6,7]. Neonatal outcomes reported in transplant recipients show a high incidence of preterm and low-birth-weight infants; more than 50% of babies born to organ transplant recipients are premature. Because breastfeeding is particularly beneficial for preterm infants, it would be an advantage for that population [1,2,8,9,10,11,12]. In the past, mothers on chronic immunosuppressive therapy (tacrolimus or cyclosporine) were advised against breastfeeding [5,6,7,13]. It is known that, after oral administration, tacrolimus reaches its peak blood levels after about 1 to 3 h, and the drug is excreted into breast milk. Therefore, according to the summary of product characteristics, its harmful effects on the newborn cannot be ruled out, and women receiving tacrolimus should not breastfeed [14]. In recent years, however, few reports emerged confirming the presence of tacrolimus in trace levels in the mother’s milk. Therefore, some authors and current guidelines of scientific societies suggest that this policy might be changed. Therefore, we designed the study aiming to assess the concentration of tacrolimus and its M-1 and M-3 metabolites in the colostrum of the post-transplant mothers and the peak level in relation to the time elapsed from the last dose of medication; to assess the amount of tacrolimus and its M-1 and M-3 metabolites that would be ingested by the neonates whose mothers would choose to breastfeed; and to assess the mean concentration of tacrolimus and its metabolites in cord blood and in the venous blood of the newborns.

## 2. Materials and Methods

The study group consisted of 14 graft recipients on tacrolimus therapy (including 13 after liver and 1 after kidney transplantation) and their neonates born in the First Department of Obstetrics and Gynecology, Medical University of Warsaw, between 2012 and 2015. All the mothers enrolled in the study signed an informed consent form. The study was approved by the Institutional Review Board (KB/195/2012). During pregnancy and after delivery, the tacrolimus concentrations in the mothers’ blood was measured, and the bi-daily dose of drug was titrated according to blood concentration to maintain a therapeutic level of 5–10 ng/mL. Preterm delivery between 30 and 36 weeks of pregnancy was observed in 64.3% of cases, and 50% of the newborns had a low birth weight of <2500 g. The selected details on the characteristics of the study group are shown in Table 1.

Samples were collected as follows: colostrum from the mother (70 samples), umbilical venous cord blood (14 samples), and venous blood of the newborn (14 samples). Colostrum samples were collected from the mothers who agreed to sustain lactation and were not administered lactation-suppressing drugs after the delivery. Every mother delivered five samples of colostrum in the amount of two milliliters each into sterile test tubes. Colostrum was expressed manually by the mothers according to a schedule in consecutive time points: 1t before the dose (directly before the next dose of immunosuppressant); 2t 2 h after the dose; 3t 4 h after the dose; 4t 6 h after the dose; and 5t 8 h after the dose. None of the mothers decided to breastfeed their infant and, after the sample collection, the mothers received lactation-suppressing drugs. All the newborn babies were fed with formula after the delivery. On the day of sample collection, the amount of formula ingested by every newborn was also registered. We assumed that it was equal to the amount of colostrum that a breast-fed neonate would ingest.

Three milliliters of umbilical cord blood were collected immediately after the delivery from the umbilical vein, with a sterile needle and syringe, into a sterile Ethylene-Diamine-Tetra-Acetic Acid (EDTA) test tube. One milliliter of the newborn’s venous blood was collected with a sterile needle and syringe into a sterile EDTA test tube on the first day of life, together with other routine blood tests that were performed.

Collected samples were used to measure the concentrations of the immunosuppressive drug tacrolimus and its metabolites 13-O-desmethyl tacrolimus (M-1) and 15-O-desmethyl tacrolimus (M-3). Metabolite 31-O-desmethyl Tacrolimus (M-2) levels were undetectable in our material.

Test material: Samples were deep frozen and transported to the Institute of Biochemistry and Biophysics of the Polish Academy of Sciences. Materials were secured and stored until the time of analysis in the low-temperature freezer (−80 °C) in the Laboratory of Mass Spectrometry, Drug and Metabolites Analysis Division.

Chemicals: Ascomycin and tacrolimus were acquired from Toronto Research Chemicals, Inc. (North York, ON, Canada). 13-O-desmethyl tacrolimus (M-1) and 31-O-desmethyl tacrolimus (M-2) were kindly provided by Astellas Pharma (Osaka, Japan). All stock solutions were prepared in methanol and stored in −20 °C. Commercial kits 6PLUS1^®^ Multilevel Calibrator Set Immunosuppressants in whole blood and Chromsystems MassCheck^®^ Immunosuppressants whole blood control was obtained from Chromsystems Instruments & Chemicals GmbH (Munich, Germany). Liquid chromatography coupled with mass spectrometry (LC-MS) grade methanol, high performance liquid chromatography (HPLC) grade methanol, HPLC grade acetonitrile, methyl-tert-butyl ether, and formic acid were purchased from J.T. Baker. Zinc sulfate monohydrate was purchased from Sigma-Aldrich (St. Louis, MO, USA), and analytical grade ammonium acetate was obtained from POCH (Gliwice, Poland). Ultra-pure water was produced by a water purification system (Milli-Q, Millipore, Milford, MA, USA).

Sample extraction procedure: Whole blood samples were prepared by the protocol described before [15]. Colostrum samples were prepared by transferring 2 mL (patient samples, calibrators, and quality control samples) to a 9-mL test tube, and a 700 µL of 2% aqueous zinc sulfate solution with internal standard (3 ng/mL) and 350 µL of acetonitrile were added. After brief vortexing, 3 mL of methyl-tert-butyl ether was added to extract tacrolimus and metabolites. Samples were vortexed for 3 min and centrifuged for 10 min at 3000 rpm. The organic portion was transferred into a clean 1.5-mL test tube and evaporated under a stream of nitrogen in a water-bath Turbo-Vap evaporator (Caliper Life Sciences, Hopkinton, MA, USA). The residue was reconstituted in 100 µL 75% methanol by vortexing 10 min and transferred into a chromatographic vial. The injection volume was 30 µL.

Analyses: The LC/MS method was followed as described before [16]. The concentration of tacrolimus and its metabolites was calculated using a calibration curve derived from a series of calibrator samples. Calibration curves for all analytes were generated by comparing the ratio of the peak area of the analyzed compound to the peak of the internal standard against known standard tacrolimus or metabolite concentrations. The peak area ratio of the patient sample was compared with an obtained calibration curve. Tacrolimus concentration was determined using calibrators from a commercially available kit, 6PLUS1^®^ Multilevel Calibrator Set Immunosuppressants in whole blood (Chromsystems, Munich, Germany). The calibration curve range for tacrolimus was 2.38–43.9 ng/mL. For M-1 and M-2 in-house calibration, samples were prepared by spiking standard stock solutions in human whole blood from volunteers with a final range of 0.01–1.5 ng/mL for both metabolites. For the determination of concentration in colostrum, own calibration standard mix was prepared for all analytes by diluting standard stock solutions of tacrolimus and metabolites in blank colostrum. The concentration range was 0.025–5 ng/mL for tacrolimus, 0.01–1.5 ng/mL for M-1, and 0.005–1.5 ng/mL for M-2. Concentration of M-3 tacrolimus was quantified using an M-1 tacrolimus calibration curve. Mean R2 coefficients of calibration curves from six calibration samples were not lower than 0.97. To ensure control of the method for the determination of analytes in whole blood, external control samples (Chromsystems MassCheck^®^ Immunosuppressants whole blood control) for tacrolimus and in-house control samples for metabolites were used. For the colostrum procedure, control samples were prepared by adding tacrolimus and metabolites into drug-free colostrum. The method showed good inter-assay and intra-assay precision below 10%.

Data concerning tacrolimus and its metabolite concentration in colostrum were analyzed to estimate the amounts that would be ingested by a breast-fed infant. The AUC parameter (area under the curve) was found by adding the area of the trapezoids method with the use of a PK package (ver. 1.3). For every mother, the mean concentration of tacrolimus and its metabolites in colostrum was calculated with use of the AUC parameter in timepoints: 1t, 2t, 3t, 4t, and 5t, repeating the measured value from t1 as a concentration after 12 h (before the next oral dose) and multiplying twice to estimate the result after 24 h. The result was divided by 24 and multiplied by the amount of milk that a breast-fed newborn would ingest on the day of sample collection. Thus, the amount of ingested tacrolimus and its metabolites for every newborn was calculated in ng/24 h and then recalculated by the dose, based on kg of bodyweight per 24 h.

All statistical analyses were performed by the R Project for statistical computing (version 3.3.2, R Foundation for Statistical Computing, Vienna, Austria). In the statistical analysis, the comparison of two independent samples was performed by a Mann–Whitney nonparametric test. The nonparametric test was used because the distribution of analyzed variables was not normal, which was confirmed by means of a Shapiro–Wilk test. The results in time were compared using the ANOVA Friedman test, which is a nonparametric counterpart of the analysis of variance for repeated measurements. A *p* value ≤ 0.05 was considered statistically significant.

## 3. Results

**Figure 1:**
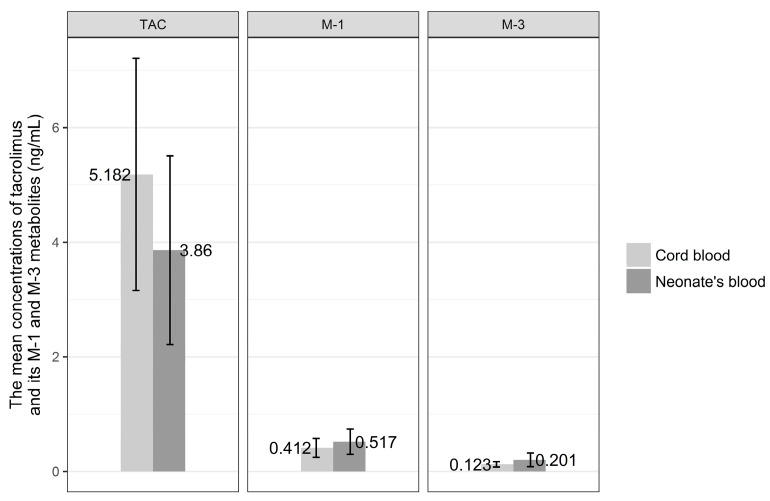
The mean concentrations of tacrolimus (TAC) and its M-1 and M-3 metabolites in colostrum collected at consecutive timepoints (1t, 2t, 3t, 4t, 5t).

The concentrations of tacrolimus and its metabolites, M-1 and M-3, were measured in colostrum collected in time points 1t, 2t, 3t, 4t, and 5t (Figure 1).

The highest concentration of tacrolimus in colostrum was observed after 8 h from oral dose and was equal to 3.219 ng/mL SD ± 2.218. Similarly, 8 h after drug intake, the mean concentration of M-1 metabolite reached peak level equal to 0.56 ng/mL SD ± 0.60. M-3 metabolite concentration in colostrum was highest after 6 h from drug intake by the mother and was 0.29 ng/mL SD ± 0.218. The lowest mean concentrations of tacrolimus and its M-1 and M-3 metabolites were noted just before the next dose of drug and were for tacrolimus 2.351 ng/mL SD ± 1.44, for M-1 metabolite 0.34 ng/mL SD ± 0.250, and 0.19 ng/mL SD ± 0.16 for M-3 metabolite.

No statistical difference was revealed when the concentrations of tacrolimus and its metabolites, M-1 and M-3, in colostrum in consecutive time points were analyzed (chi^2^; *p* = 0.099, *p* = 0.305, *p* = 0.729, respectively).

The amount of formula ingested by the newborns on the day of colostrum collection and estimated amounts of tacrolimus and its metabolites, M-1 and M-3, ingested with colostrum converted into dose by kilogram of body weight per 24 h are presented in Table 2.

**Figure 2:**
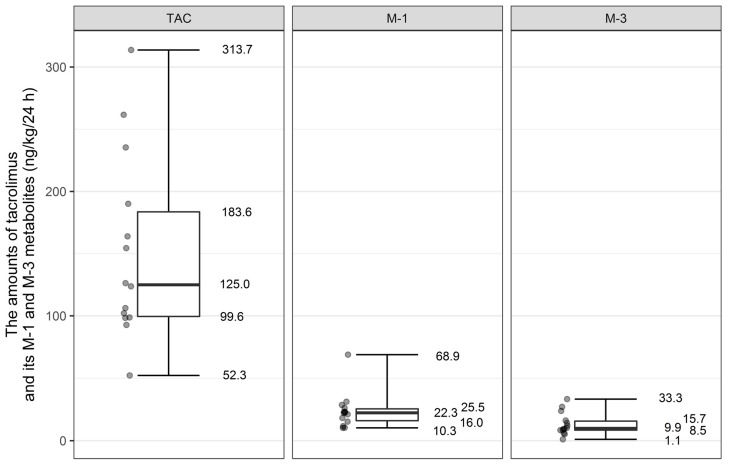
Distribution of tacrolimus and its metabolites M-1 and M-3 amount that would be ingested by breast-fed infants (ng/kg/24 h).

In our group, the mean dose of tacrolimus that would be ingested by the breast-fed infants was 151.4 ng/kg/24 h (SD ± 74.39). The doses of metabolites were 23.80 ng/kg/24 h (SD ± 14.53) for M-1 and 13.25 ng/kg/24 h (SD ± 9.05) for M-3 metabolite (Figure 2).

We estimated amounts of tacrolimus and its metabolites (in ng/kg/24 h) that would be ingested by LBW infants in comparison to those born with body weight above 2500 g. Mean dose of tacrolimus and M-3 metabolite that would be ingested by LBW newborns would be similar to that by newborns weighing > 2500 g (for tacrolimus: 143.05 ng/kg/24 h (SD ± 78.33) vs. 159.75 ng/kg/24 h (SD ± 75.44); *p* value = 0.54 and for M-3 metabolite: 13.81 ng/kg/24 h (SD ± 11.22) versus 12.68 ng/kg/24 h (SD ± 7.14); *p* value 0.12). The statistical significance was noted only in the case of M-1 metabolite. The amount of ingested M-1 metabolite would be on average higher in babies born with body weight above 2500 g in comparison to LBW infants (30.42 ng/kg/24 h (SD ± 17.72) versus 17.19 ng/kg/24 h (SD ± 6.43); *p* value = 0.03).

The amount of tacrolimus and its metabolites ingested by the infants born prematurely (<37 weeks of gestation) and term infants was also subject to analysis. There was no difference in mean values of tacrolimus and its M-3 metabolite that would be ingested by the premature infants in comparison to those born at term (for tacrolimus 136.23 ng/kg/24 h (SD ± 69.52) vs. 178.70 (SD ± 82.87); *p* value = 0.24; for M-3 metabolite 12.44 ng/kg/24 h (SD ± 10.12) vs. 14.697 (SD ± 7.57); *p* value = 0.438). The amount of ingested M-1 metabolite would be on average higher in babies born at term in comparison to infants born prematurely (114.28 (SD ± 102.77) vs. 45.50 (SD ± 23.93); *p* value = 0.042).

**Figure 3:**
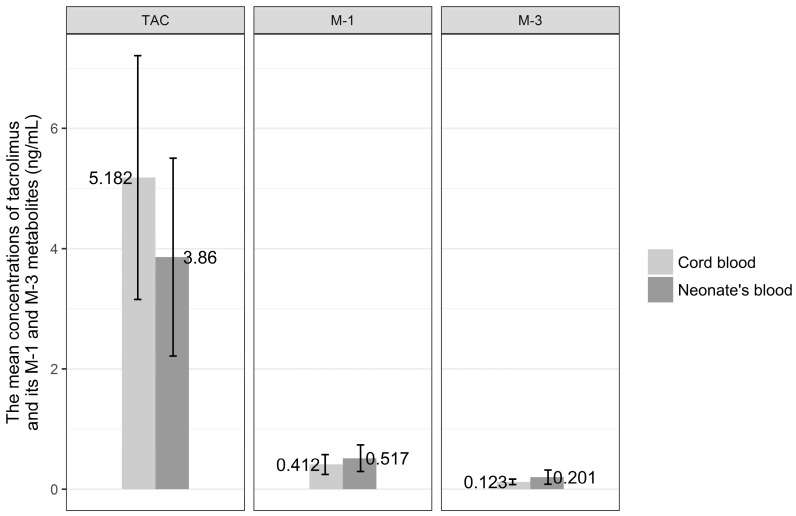
The mean concentration of tacrolimus (TAC) and its M-1 and M-3 metabolites in venous cord blood and in venous blood of the newborns.

The distributions of the concentrations of tacrolimus and its metabolites in venous cord blood and in venous blood of the neonates are presented in detail in Figure 3. The mean concentration of tacrolimus in cord blood was 5.20 ± 1.94 ng/mL, the M-1 metabolite was 0.41 ± 0.168 ng/mL, and M-3 metabolite was 0.12 ± 0.46 ng/mL. The mean concentration of tacrolimus in venous blood of the newborn was 3.89 ± 1.58 ng/mL, the M-1 metabolite was 0.51 ± 0.21 ng/mL, and the M-3 metabolite was 0.20 ± 0.11 ng/mL.

Mean concentration of tacrolimus in cord blood was higher than that in venous blood of the newborn, whereas the metabolite concentrations were higher in venous than in cord blood, but the comparison of mean concentrations of tacrolimus and its metabolites, M-1 and M-3, in venous cord blood and in venous blood of the newborn revealed no statistical difference.

The correlation coefficient between mean concentration of tacrolimus and its metabolites, M-1 and M-3, in colostrum and their concentrations in cord blood were calculated, and the analysis revealed no statistical difference.

## 4. Discussion

The important aspect of our study was to estimate the amount of tacrolimus and its metabolites, M-1 and M-3, that would be ingested by the newborns during the first days of life if the mothers chose to breastfeed. The results show that the amounts would be very small both for tacrolimus (0.15 µg/kg/24 h) and for its metabolites, M-1 and M-3 (0.02 and 0.01 µg/kg/24 h, respectively). The measurement of tacrolimus and its metabolite concentration in 70 samples of colostrum collected from 14 post-transplant mothers allowed for the acquisition of a relatively large data set and complements limited data published in medical databases. The limitation of our study is mainly due to the small number of participants and the fact that post-transplant mothers did not choose to breastfeed and just sustained lactation for a few days after the delivery to collect colostrum samples for our study. Colostrum is different from mature breast milk in its composition and volume. Therefore, the limitation of our study is to assume the amount of excreted tacrolimus and its metabolites in colostrum exactly equals that excreted in mature breast milk. Unfortunately, the participating mothers did not wish to sustain lactation for longer periods of time. Therefore, designing a study that would assess the safety of breastfeeding in larger groups of post-transplant mothers and sustaining lactation for a longer period of time would be very difficult in practice.

The high percentage of preterm births among posttransplant mothers (20% of kidney graft recipients deliver before 34 weeks and 50% before 37 weeks of pregnancy and for liver recipient these numbers reach 39% and 16% respectively) is confirmed in the recently published Annual Report of Transplant Pregnancy Registry. Similarly, the prevalence of low birth weight (LBW) and very low birth weight (VLBW) neonates reaches 42% and 10% in kidney graft pregnancies and 30% and 8% in mothers after liver transplantation [17]. Therefore, in the light of published data on the risks and benefits of breastfeeding, breastfeeding seems to be especially important in this subgroup of neonates born to graft recipients. Early enteral milk feeding significantly reduces the risk of necrotizing enterocolitis in LBW and preterm infants [18,19]. ESPGHAN (The European Society for Paediatric Gastroenterology Hepatology and Nutrition) guidelines suggest that in preterm infants where no own mother’s milk is available donor human milk should be regarded as the first alternative. Unfortunately, this intervention is not feasible in every setting [20].

Currently, there are no standards regarding the feeding of neonates and infants by recipient mothers. The consequences of even minimal fetal exposure to chronic immunosuppressive therapy are unknown. However, evidence to date suggests that babies’ exposure to the drug during breastfeeding is relatively insignificant in comparison to that in utero. Some authors suggest that women taking tacrolimus who wish to breastfeed after appropriate counseling should not be discouraged from doing so. However, experts say that deciding to breastfeed while continuing treatment with an agent for which in utero exposure also has occurred is different from initiating a new therapy while breastfeeding [21,22,23].

The British Society for Rheumatology (BSR) and British Health Professionals in Rheumatology (BHPR) and the European League Against Rheumatism (EULAR) published guidelines for tacrolimus in pregnancy and breastfeeding. According to BSR, BHPR, and EULAR, tacrolimus is compatible throughout pregnancy and breastfeeding [24,25].

In 1997, Jain et al. analyzed the concentrations of tacrolimus in mother’s blood, colostrum, placenta, cord blood, and baby’s blood. The authors suggested that the infants were protected from drug toxicity in utero by a partial placental barrier. In 9 of 10 placental samples, the tacrolimus concentration was 2 to 56 times higher than that in cord blood. Placental tacrolimus levels also usually exceeded that of maternal plasma (median fourfold). Tacrolimus was present in all 10 breast milk samples from six mothers at almost half the concentration as in contemporaneous maternal samples (median ratio was 0.5). The mean tacrolimus concentration in the colostrum was 0.79 ng/mL ± 0.48. None of the mothers chose to breastfeed [26].

In our study, the mean concentration of tacrolimus measured in 70 samples of colostrum was four times higher. The peak level (3.23 ng/mL, SD ± 2.218) was observed after 8 h from oral dose, and the lowest concentration (2.35 ng/mL, SD ± 1.44) was noted just before the next dose of tacrolimus. Jain et al. did not analyze the metabolite concentrations.

French et al. published a case report based on one liver recipient mother, suggesting that maternal therapy with tacrolimus may be compatible with breastfeeding [27]. The authors reported tacrolimus concentrations in human milk while the mother was exclusively breastfeeding the infant. The mean concentration of tacrolimus in the milk was 0.43 ng/mL. The highest concentration of tacrolimus in milk (0.57 ng/mL) was observed one hour after oral dose. Therefore, the tacrolimus dose that an exclusively breast-fed infant would ingest was 0.06 μg/kg/24 h (0.43 ng/mL × 150 mL/kg/24 h), which is 0.06% of the mother’s weight-adjusted dose (0.1 mg/kg/24 h). The maximum tacrolimus dose the infant would receive through breastfeeding was 0.09 μg/kg/24 h, which corresponds to about 0.09% of the mother’s weight-adjusted dose.

Our study suggests that the maximal concentration of tacrolimus in colostrum was more than 5 times higher than in the study by French et al. (3.22 ng/mL versus 0.57 ng/mL) and was noted 8 h after the oral dose.

The case report by Gardiner et al. shows that the average tacrolimus concentration in milk was 1.8 µg/L, and the baby ingested approximately 0.5% of the maternal dose (weight-adjusted). The highest concentration of tacrolimus in milk (2.1 µg/L) was observed 8.5 h after the maternal dose [28]; this finding was similar to our result.

In 2013, Bramham et al. compared tacrolimus levels in breast-fed and formula-fed infants, which did not reveal any differences, and the blood concentrations decreased with the time elapsing from delivery. In this study, the estimated infant dose was based on the highest breast milk concentration [22]. Bramham et al. assumed that a neonate would drink 0.15 L/kg/24 h of breast milk; for a neonate weighing 2.4 kg, that would be 0.36 L/24 h of breast milk. Considering that the maximal concentration of tacrolimus in breast milk was 1.56 µg/L, it was estimated that the neonate would ingest 0.24 µg/kg/24 h.

In our study, we precisely measured the amount of milk taken by every newborn on the day of collection of colostrum samples and the concentration of tacrolimus in colostrum of every patient. Our analysis indicates that the newborns would on average ingest 0.15 µg/kg/24 h of tacrolimus with breast milk, which is less than in the study by Bramham et al., although the mean maximal concentration of tacrolimus was higher in our study than in the above-cited study by Bramham et al. (3.22 µg/L versus 1.56 µg/L). The difference may be explained by the fact that our patients collected only colostrum samples, whereas in the study by Bramham et al. the breast milk samples collected between days 1 and 72 were analyzed. Nevertheless, the authors did not observe any relationship between breast milk concentration and number of days postpartum.

According to Bramham et al., low birth weight at delivery was significantly associated with higher infant tacrolimus levels. Infant tacrolimus levels fell with higher birth weights; for each 500-g increase, there was a −25% change in geometric mean tacrolimus level (95% confidence interval CI, −42% to −1%; *p* = 0.034). In our study, there were no such associations. There was no difference in the average amount of tacrolimus that infants would ingest with birthweight < 2500 g (143.05 ng/kg/24 h) and ≥2500 g (159.75 ng/kg/24 h).

In the study by Zheng et al., peak and trough concentrations in milk were 1.11 and 0.78 ng/mL, respectively. The mean concentration of tacrolimus in milk was 0.93 ng/mL. Daily tacrolimus excretion into milk was estimated to be 32.0 ng. The amount of tacrolimus excreted into breast milk over a 12 h, steady-state dosing interval was determined in only one patient, treated with 1.5 mg of oral tacrolimus twice daily. Tacrolimus concentration in breast milk peaked later than in blood or plasma [29].

The data from the National Transplantation Pregnancy Registry, encompassing the largest case series, also show that we might be cautiously optimistic about the immunosuppressant use in breastfeeding females. Other investigators and clinicians suggest that breastfeeding by transplant recipient mothers remains an area in which recommendations are evolving; future research should include studies that lead to the establishment of guidelines for dosing, the measurement of levels of drugs, and the long-term follow-up of children breast-fed by recipients on maintenance immunosuppressive therapy. The preliminary findings are promising [2,12,21,24,25,29,30,31,32,33,34].

## 5. Conclusions

Low concentrations of tacrolimus and its metabolites, M-1 (13-O-desmethyl tacrolimus) and M-3 (15-O-desmethyl-tacrolimus), in colostrum indicate that breast-fed neonates will ingest trace amounts of drugs, thus presenting the possibility that neonates could be safely breast-fed. Our results show that a breast-fed neonate would ingest 150 ng/kg/24 h and, assuming maternal dose being between 0.1 to 0.2 mg/kg/24 h, this means the infant’s dose would be approximately 1/1000th that of the mother’s.

According to a recently published Annual Report of Transplant Pregnancy Registry International, there is a notable increasing trend in the number of graft recipients choosing to breastfeed, with the number exceeding 60% of live births in 2016 [17]. It is necessary to gather more data to confirm the safety of breastfeeding by posttransplant mothers, but the exposure through breastmilk seems to be minimal. In light of the findings of these data, the possibility of breastfeeding by these patients will be an extremely important element in preventing lifestyle diseases and obesity in children and will improve the health of the baby. Undoubtedly, the possibility of breastfeeding would also be a very precious experience for organ transplant mothers, allowing for the full enjoyment of motherhood, which could have a very positive impact on the struggle with their underlying disease. The possibility of breastfeeding could indicate their full social and medical recovery after a successful organ transplantation.

## Figures and Tables

**Table 1:** Selected details on study group characteristics.

Patient No.	1	2	3	4	5	6	7	8	9	10	11	12	13	14
Years from transplant	9	3	3	11	6	13	5	2	5	11	4	7	11	4
Liver (LT) or Kidney Graft (KT) and underlying disease	LT AIH	LT Hep B	LT WD	LT WD	LT Hep C	LT WD	LT AIH	LT PSC	KT GN	LT WD	LT AIH	LT	LT HCa	LT AIH
Other diseases	RCh	-	Ch	-	-	DM	-	CU	HA	DM	-	-	HA	-
Mode of delivery	CS	VD	CS	CS	CS	CS	CS	CS	VD	CS	VD	VD	CS	CS
Weeks of pregnancy	30	36	34	38	36	39	34	39	36	34	32	39	33	38
Birth weight (g)	1320	3090	2280	3250	2900	3120	2350	3400	2400	2450	2010	4300	1870	2610
Tacrolimus dose (mg)	2 + 2	3 + 2	3 + 2	7 + 7	7 + 7	5 + 4	3 + 3	4 + 3	5 + 4	3 + 3	5 + 5	2 + 3	3 + 3	3 + 2
Other IS drugs	-	-	AZA, P	-	AZA, P	-	P	P	AZA, P	P	P	AZA, P	P	AZA, P

Abbreviations: LT: liver transplant, KT: kidney transplant, AIH: autoimmune hepatitis, Hep B: Hepatitis B, Hep C: hepatitis C, WD: Wilson disease, GN: glomerulonephritis, HCa: hepatocellular carcinoma, RCh: recurrent cholangitis, Ch: cholestasis, CU: ulcerative colitis, DM: diabetes mellitus, HA: arterial hypertension, CS: cesarean section, VD: vaginal delivery, P: prednisone, AZA: azathioprine.

**Table 2:** Characteristics of neonates and amounts of tacrolimus and its metabolites potentially ingested with breastmilk.

Patient No.	Weeks of Pregnancy	Birthweight (g)	Day of Colostrum Collection	Amount of Ingested Formula (mL)	Amount of Tacrolimus (ng/24 h)	Dose of Tacrolimus (ng/kg/24 h)	Amount of M-1 (ng/24 h)	Dose of M-1 (ng/kg/24 h)	Amount of M-3 (ng/24 h)	Dose of M-3 (ng/kg/24 h)
1	30	1320	4	70	68.979	52.257	29.342	22.229	1.517	1.149
2	36	3090	2	160	304.013	98.386	71.453	23.124	18.453	5.972
3	34	2280	2	110	242.284	106.265	59.904	26.274	12.247	5.371
4	38	3250	3	240	532.74	163.92	72.86	22.418	27.56	8.48
5	36	2900	2	140	366.333	126.322	90.615	31.247	27.067	9.333
6	39	3120	2	160	385.907	123.688	89.4	28.654	37.733	12.094
7	34	2350	2	140	614.798	261.616	42.782	18.205	56.058	23.855
8	39	3400	2	160	347.12	102.094	52.027	15.302	32.507	9.561
9	36	2400	2	140	564.912	235.38	28.105	11.71	79.998	33.333
10	34	2450	2	120	241.84	98.71	25.23	10.298	20.89	8.527
11	32	2010	2	120	186.35	92.711	42.52	21.154	28.61	14.234
12	39	4300	2	180	1348.89	313.695	296.4	68.93	116.775	27.157
13	33	1870	3	120	288.8	154.439	19.53	10.444	19.04	10.182
14	38	2610	3	200	496.2	190.115	60.717	23.263	42.267	16.194
